# Imbalance in Fatty-Acid-Chain Length of Gangliosides Triggers Alzheimer Amyloid Deposition in the Precuneus

**DOI:** 10.1371/journal.pone.0121356

**Published:** 2015-03-23

**Authors:** Naoto Oikawa, Teruhiko Matsubara, Ryoto Fukuda, Hanaki Yasumori, Hiroyuki Hatsuta, Shigeo Murayama, Toshinori Sato, Akemi Suzuki, Katsuhiko Yanagisawa

**Affiliations:** 1 Department of Drug Discovery, Center for Development of Advanced Medicine for Dementia, National Center for Geriatrics and Gerontology, Aichi, Japan; 2 Department of Bioscience and Informatics, Keio University, Kanagawa, Japan; 3 Department of Neuropathology, Tokyo Metropolitan Institute of Gerontology, Tokyo, Japan; 4 Institute of Glycoscience, Tokai University, Kanagawa, Japan; 5 Department of Alzheimer’s Disease Research, Center for Development of Advanced Medicine for Dementia, National Center for Geriatrics and Gerontology, Aichi, Japan; Weizmann Institute of Science, ISRAEL

## Abstract

Amyloid deposition, a crucial event of Alzheimer’s disease (AD), emerges in distinct brain regions. A key question is what triggers the assembly of the monomeric amyloid ß-protein (Aß) into fibrils in the regions. On the basis of our previous findings that gangliosides facilitate the initiation of Aß assembly at presynaptic neuritic terminals, we investigated how lipids, including gangliosides, cholesterol and sphingomyelin, extracted from synaptic plasma membranes (SPMs) isolated from autopsy brains were involved in the Aß assembly. We focused on two regions of the cerebral cortex; precuneus and calcarine cortex, one of the most vulnerable and one of the most resistant regions to amyloid deposition, respectively. Here, we show that lipids extracted from SPMs isolated from the amyloid-bearing precuneus, but neither the amyloid-free precuneus nor the calcarine cortex, markedly accelerate the Aß assembly *in vitro*. Through liquid chromatography-mass spectrometry of the lipids, we identified an increase in the ratio of the level of GD1b-ganglioside containing C20:0 fatty acid to that containing C18:0 as a cause of the enhanced Aß assembly in the precuneus. Our results suggest that the local glycolipid environment play a critical role in the initiation of Alzheimer amyloid deposition.

## Introduction

Alzheimer’s disease (AD) is the most common and devastating dementia that ultimately causes death. The early and invariable neuropathological hallmark of AD is the deposition of the amyloid ß-protein (Aß) as fibrils (amyloid). Besides its direct toxicities, amyloid can also act as a potential reservoir [1–3] and/or generator [4] of Aß oligomers, which are the prime synaptotoxic agents in AD brains [5]. It is widely accepted that amyloid deposition is a consequence of chronic imbalance between Aß production and Aß clearance [6]; however, the mechanism underlying the initiation of the assembly of monomeric Aß into fibrils, that emerges in distinct brain regions of the brain, remains to be elucidated.

We previously examined autopsied brains and discovered the GM1-ganglioside-bound Aß (GAß) in a brain with early pathological changes of AD [7]. On the basis of the unique molecular characteristics of GAß such as its high potency to facilitate Aß assembly [8,9], we hypothesized that GAß is an endogenous seed for Alzheimer amyloid in the brain [9]. To date, a body of evidence has been growing to support the GAß hypothesis [10–14]. Notably, we have recently succeeded in the detection of GAß in the brain of a young transgenic mouse AD model before amyloid deposition [15]. Nevertheless, the hypothesis has been challenged by a simple question of how GAß is favorably generated in particular brain regions that are prone to amyloid deposition.

To elucidate this issue, we re-examined autopsied brains by focusing on the precuneus, one of the most vulnerable region to amyloid deposition and also an implicated region for early cognitive dysfunction of AD [16–18], comparing it with the calcarine cortex, one of the most resistant region to amyloid deposition [19]. Given that GAß generation exclusively depends on the lipid environment of host membranes [20,21] and most likely occurs at presynaptic neuritic terminals through selective alteration in the lipid composition [22,23], we designed the study as follows. First, to search for lipid alterations that are responsible for the early amyloid deposition, we investigated the precuneus bearing amyloid at only modest level, and compared it with the amyloid-free precuneus and the calcarine cortex of each brain. Second, to obtain information on the lipid composition linked to GAß generation, we analyzed the lipids extracted from synaptic plasma membranes (SPMs) isolated from the cortical regions of interest. Third, to eliminate the influence of the apolipoprotein E genotype of individuals, which is a strong modulator of amyloid deposition and likely affects the lipid composition of SPMs, the cases analyzed were restricted to genotype ε3/ε3 except for one case with ε3/ε4.

## Materials and methods

### Tissue source

Specimens of the human precuneus and calcarine cortex were obtained from the Brain Bank for Aging Research at Tokyo Metropolitan Institute of Gerontology (http://www.mci.gr.jp/BrainBank/index.cgi) with the approval of the Ethics Committees of the National Center for Geriatrics and Gerontology and Tokyo Metropolitan Institute of Gerontology. The specimens were neuropathologically characterized by modified methenamine, Gallyas-Braak silver staining, and immunohistochemical staining using anti-Aß 11–28 (12B2, monoclonal; IBL, Maebashi, Japan) and anti-phosphorylated tau (AT8, monoclonal; Innogenetics, Temse, Belgium) antibodies as previously reported [24]. The human brain specimens were neuropathologically classified in accordance with the criteria of Braak and Braak [19] and the Consortium to Establish a Registry for Alzheimer Disease (CERAD) [25]. The clinical dementia rating scale (CDR) [26] was retrospectively determined by two independent board-certified neurologists. Demographics and characteristics of the autopsied brains used in this study are shown in Table 1. No significant difference was observed in the age of death, CDR, Braak neurofibrillary tangle (NFT) stage, and the post-mortem delay between amyloid-free and amyloid-bearing subjects. The apolipoprotein E genotype of the all subjects analyzed in this study was ε3/ε3 except one subject, ε3/ε4, in the amyloid-bearing group. There were no significant differences between the two groups in terms of any of the demographic neuropsychiatric measures analyzed in this study.

**Table 1:** Demographics and characteristics of the autopsied brains.

	Age (y)/ gender	CDR	BraakSP stage	Neuritic plaque density (CERAD)	Braak NFT stage	PMD (h:min)	Cause of death
**Amyloid free**	64/M	0	0	0	I	2:55	Leukemia
	72/M	0	0	0	II	17:22	Liver cirrhosis
	74/M	0	0	0	I	14:18	Peritonitis
	74/F	0	0	0	I	15:40	Pneumonia
	74/M	0.5	0	0	I	16:18	Multiple myeloma
	77/M	1	0	0	I	2:15	Chronic myocarditis
	77/M	1	0	0	II	11:04	Sepsis
	78/F	3	0	0	I	1:56	Pancreatitis
	85/F	3	0	0	I	20:40	Myocardial infarction
	89/F	3	0	0	III	14:43	Lymphoma
	94/F	0	0	0	II	3:29	DIC
Mean or median	78.0 ± 8.4	0.5	0	0	I	11.0 ± 7.0	
**Amyloid bearing**	69/M	0	A	1	I	14:52	Acute renal failure
	74/M	0	A	2	I	12:11	Pneumonia
	76/M	N/A	A	1	II	18:10	Pneumonia
	80/M	0	A	1	II	8:50	Hepatic cancer
	80/M	1	A	1	I	7:57	Pneumonia
	78/F	0	A	0	I	17:15	Lymphoma
	84/F	3	A	0	I	4:25	Pneumonia
	90/M	1	A	1	I	8:10	Pneumonia
	91/M	3	A	1	II	8:24	Pneumonia
	93/F	0.5	A	2	II	7:42	Cholecystitis
Mean or median	81.5 ± 7.9	0.5	A	1	I	10.8 ± 4.6	

Mean ± SD (Age and PMD) or median (CDR, Braak SP stage, neuritic plaque density, and Braak NFT stage) are shown.

CDR, clinical dementia rating scale; SP, senile plaque; CERAD, Consortium to Establish a Registry for Alzheimer Disease; NFT, neurofibrillary tangle; PMD, post-mortem delay; DIC, disseminated intravascular coagulation; N/A, not available.

### Preparation of synaptosomes

Synaptosomes were prepared as previously reported [27]. Briefly, after homogenization of the gray matter in ice-cold buffer A (10 mM HEPES, 0.32 M sucrose, 0.25 mM EDTA, pH 7.4), postnuclear supernatant (PNS) was collected by centrifugation at 580 × *g* for 8 min at 4°C. The resultant crude mitochondrial pellet (CMP) was collected from PNS by recentrifugation at 14,600 × *g* for 20 min at 4°C. The CMP was suspended in buffer B (10 mM HEPES, 0.32 M sucrose, pH 7.4) by hand homogenization, layered over 7.5% and 14% Ficoll in buffer B, and then centrifuged at 87,000 × *g* for 30 min at 4°C. The interface between 7.5% and 14% Ficoll solutions rich in synaptosomes was collected.

### Preparation of synaptic plasma membranes (SPMs)

SPMs were prepared as previously reported [27]. Briefly, synaptosomes were osmotically shocked in ice-cold 5 mM Tris buffer (pH 8.5) by stirring on ice with occasional vortex mixing. After centrifugation at 43,700 × *g* for 20 min at 4°C, the collected crude SPM pellet was suspended in buffer B, layered over 25% and 32.5% sucrose in 10 mM HEPES (pH 7.4), and then centrifuged at 41,000 × *g* for 30 min at 4°C. The interface between 25% and 32.5% sucrose solutions rich in SPMs was collected.

### Measurement of cholesterol level

Cholesterol level was measured using an Amplex-Red cholesterol assay kit (Life Technologies, Carlsbad, CA) as previously reported [28].

### Lipid extraction and GD1b-gangliosides purification

Lipids were extracted as previously reported [27]. Briefly, total lipids were extracted from collected SPMs with chloroform-methanol (2:1; v/v) and chloroform-methanol-water (1:2:0.8; v/v/v). Gangliosides and sphingomyelin were separately collected by two-phase partition [29]. The collected upper phase containing gangliosides and the lower phase containing sphingomyelin were used for liquid chromatography-mass spectrometry (LC-MS). Synthetic GM1(d18:1–^13^C16:0) (Tokyo Chemical Industry, Tokyo, Japan) and sphingomyelin [SM(d18:1–17:0)] (Avanti Polar Lipids, Alabaster, AL) were added to upper phase and lower phase, respectively, and used as the internal standards for LC-MS. GD1b(d20:1–20:0) and GD1b(d20:1–18:0) were purified from porcine brain GD1b-gangliosides (Avanti Polar Lipids) by HPLC with a Develosil C30 column (4.6 mm × 250 mm, Nomura Chemical Co) and a gradient elution made with solvent A [5 mM ammonium formate in water-methanol (1.25:98.75, v/v)] and solvent B [5 mM ammonium fornate in water-methanol-isopropanol (1.25:48.75:50, v/v)]. Programmed elution was performed as follows: from 0% to 100% B for 25 min, 100% B for 15 min, and flow rate at 1ml/ min. Fractionated effluents were checked by LC-MS with the conditions described in the following paragraph and the amount of each purified GD1b-ganglioside was determined by LC-MS using GM1(d18:1–^13^C16:0) as the internal standard.

### Liquid chromatography-mass spectrometry (LC-MS)

Gangliosides were analyzed by LC-MS. In the analysis of the ceramide structure of gangliosides, gangliosides were separated by LC using a Develosil C30 column (1 mm i.d. × 50 mm; Nomura Chemical Co) as previously reported [27,30]. In the analysis of the glycan structure of gangliosides, gangliosides were separated by LC using an Inertsil NH_2_ column (1 mm i.d. × 50 mm; GL Sciences) and elution solvents: solvent A, 1 mM ammonium formate in water-acetonitrile (17:83 v/v); solvent B, 50 mM ammonium formate in water-acetonitrile (50:50, v/v). Gradient elution was performed as follows: 0% B in A for 5 min, from 0% to 76% B in A for 15 min, from 76% to 90% B in A for 5 min, 90% B in A for 10 min [31]. Shimadzu LC-IT-MS was used in the negative-ion- and auto-mode with the mass range from m/z 200 to 2,000 and at a detector voltage of 1.9 kV. The ratio of the intensity of ganglioside signals to that of internal standard signals was measured using mass chromatograms monitored for [M–H]^-^ ions. Ganglioside structures were confirmed by MS^2^ with the collision-induced dissociation (CID) energy at 50% arbitrary, and ceramide structures were characterized by MS^3^ or MS^4^ with manual mode detection and monitoring of corresponding m/z values.

Sphingomyelin was analyzed after alkaline treatment of the lower phase to hydrolyze glycerolipids. The lower-phase lipids were incubated in 0.1 N NaOH in methanol at 37°C for 2 hr. Then, sphingomyelin was recovered in the lower phase of Folch’s partition, after neutralizing the treated solutions with 0.1 N acetic acid. The collected sphingomyelin was analyzed by LC-MS using a Develosil C30 column (1 mm i.d. × 50 mm; Nomura Chemical Co) and solvents: solvent A, acetic acid-25% ammonia-water-methanol-isopropyl alcohol (0.1: 0.1: 20: 30: 50, v/v); solvent B, acetic acid-25% ammonia-water methanol-isopropyl alcohol (0.1: 0.1: 2: 48: 50, v/v) under a gradient elution condition (0% B in A for 5 min, from 0% to 100% B in A for 30 min, and 100% B for 5 min) and at a flow rate of 50 ml/min. Shimadzu LC-IT-MS was used in the negative-ion- and auto-mode with the mass range from m/z 500 to 1,000 at a detector voltage of 1.94 kV. The ratio of the intensity of sphingomyelin signals to that of internal standard signals was measured using MS^1^ mass chromatograms monitored for [M–H]^-^ ions. Their ceramide structures were characterized by MS^2^ with the automode monitoring of [M – H − 60]^−^ and detection of signals at m/z 449.32 and 477.34 for fragment ions derived from d18:1 and d20:1, respectively, with CID energy at 50% arbitrary. All the organic solvents used in LS-MS are of LC-MS grade (Fluka, Sigma-Aldrich, St. Louis, MO)

### AFM of the reconstituted lipid bilayer composed of SPM lipids

**Fig 1:**
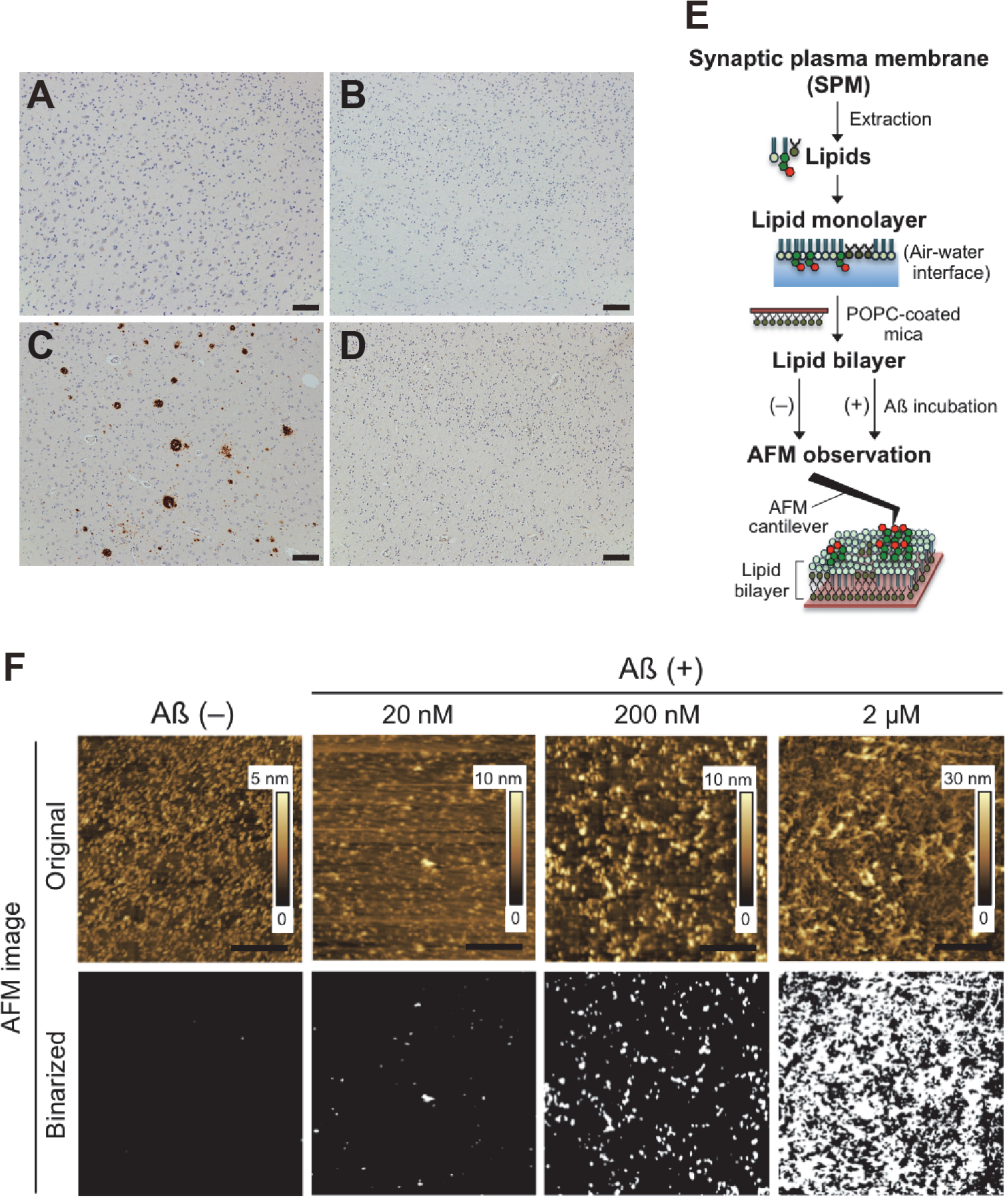
Aß assembly on reconstituted membrane of lipids extracted from SPMs isolated from autopsied brains. A-D, Representative immunohistochemical staining of the amyloid-free precuneus (A), the calcarine cortex of the brain providing A (B), the amyloid-bearing precuneus (C) and the calcarine cortex of the brain providing C (D). E, Procedure of AFM of the reconstituted lipid bilayer composed of SPM lipids. The monolayer of SPM lipids was transferred to the POPC-coated mica by horizontal deposition (the reconstituted lipid bilayer). The lipid bilayer was incubated with Aß42, and then the surface topography of the bilayer was imaged by AFM. F, Concentration-dependent Aß assembly on reconstituted membranes prepared from lipids extracted from the amyloid-bearing precuneus. Surface topographic studies of lipid bilayer were performed by AFM using SPM lipids extracted from the amyloid-bearing precuneus. AFM images were taken after Aß incubation at 20 nM, 200 nM, and 2 μM for 12 hr. To illuminate the area of Aß accumulation, original AFM images (*upper*) were binarized on the basis of the heights of membrane before its incubation with Aß42. White pixels in binarized AFM images (*lower*) were identified as Aß accumulation. Scale bar, 100 μm (A–D), 500 nm (E).

AFM was performed on combined samples in each of the four groups. Lipids were extracted from combined SPMs derived from six specimens in each group unless otherwise indicated. The bilayers composed of SPM lipids were prepared as reported previously (Fig. 1E) [32,33]. To construct the lipid bilayer, a lipid monolayer at an air-water interface was prepared on a Langmuir-Blodgett trough at 25°C (FSD-220, USI System Co., Ltd., Japan). First, a 1-palmitoyl-2-oleoyl-*sn*-glycelo-3-phosphocholine (POPC) monolayer was prepared using Milli-Q water as the subphase and transferred to the freshly cleaved mica by horizontal deposition at a surface pressure of 35 mN m^-1^ (POPC-coated mica). After drying overnight, another monolayer of SPM lipids was transferred to the POPC-coated mica by horizontal deposition at a surface pressure of 30 mN m^-1^ using phosphate-buffered saline (PBS) as the subphase (reconstituted lipid bilayer membrane). The lipid bilayer was incubated with Aß and/or antibodies in PBS for 15 min. Prior to AFM measurements, the lipid bilayer on mica was washed with PBS and then subjected to AFM.

AFM measurements were carried out on a SPM-9600 (Shimadzu, Japan) in the water phase at 25°C. A cantilever with integrated pyramidal silicon nitride tips and a spring constant of 0.1 N m^-1^ (code BL-AC40TS-C2, Olympus) was used for imaging in the dynamic mode at a scanning rate in the range of 1–2 Hz. Typically, several AFM images with a scan area of 4 μm^2^ (2 μm × 2 μm) were obtained, and the heights of the surface topography of the membrane were indicated by color bars. To estimate the area of Aß accumulation, AFM images were binarized on the basis of the heights of a bare membrane (typically 6 nm threshold), and pixels were counted using the GNU Image Manipulation Program (GIMP Portable, PortableApps.com) or ImageJ (NIH) software. Basically, a white area of the binarized image obtained was identified as the area of Aß accumulation.

### Preparation of seed-free Aß solutions

Seed-free Aß solutions were prepared as previously reported [9,34]. Briefly, synthetic Aß42 (Peptide Institute, Osaka, Japan) was dissolved in 0.02% ammonia solution at 280 μM on ice. After ultracentrifugation of the solution at 435,680 × *g* for 3 hr at 4°C, the upper one-third of the supernatant was collected and used as a seed-free Aß solution. The collected Aß solutions were stored at −80°C until use. Immediately before use, the aliquots were thawed and diluted in Tris-buffered saline (TBS) (10 mM Tris-HCl, 150 mM NaCl, pH 7.4) or PBS.

### Preparation of liposomes

Liposomes were prepared by sonication as previously reported [9] with some modifications. Briefly, after evaporation of the extracted whole lipids under N_2_ gas, the residual lipid film was hydrated and suspended in TBS by freezing and thawing. After centrifugation of the lipid suspension at 15,000 × *g* for 15 min, the resultant pellet was resuspended in TBS and sonicated for a total of 20 min (10 min, 5 min, 5 min) with cooling on ice using a probe-type sonicator (UD201, TOMY SEIKO, Tokyo). Metal debris from the titanium tip of the probe was removed by centrifugation at 3,000 × *g* for 5 min at 4°C.

### Aß assembly formation with liposomes

A seed-free Aß solution was incubated with liposomes prepared from lipids extracted from SPMs at 37°C. The protein concentration ratio of Aß to liposome was 3 μM to 0.03 μg/μl. For the analysis of amyloid fibril formation, the incubated mixture was centrifuged at 435,680 × *g* for 20 min at 4°C. The resultant pellet was washed twice with TBS and then solubilized in formic acid. After centrifugation at 20,400 × *g* for 15 min, the upper two-thirds of the supernatant was collected and dried using a centrifugal concentrator. The dried materials were dissolved in SDS-PAGE sample buffer including 8 M urea (62.5 mM Tris-HCl, 2% SDS, 10% glycerol, 5% 2-mercaptoethanol, 8 M urea, pH 6.8), and then processed for SDS-PAGE and Western blotting. For the analysis of soluble Aß, the supernatant collected by ultracentrifugation was resolved in SDS-PAGE sample buffer without 8 M urea.

### SDS-PAGE and Western blotting

SDS-PAGE and Western blotting in the analyses of amyloid fibril and soluble Aß were performed as previously reported [28]. Briefly, the samples dissolved in SDS-PAGE sample buffer were subjected to PAGE using 4–20% gradient gel and Tris/tricine buffer (both Cosmo Bio, Tokyo, Japan). The separated proteins were electrotransferred onto a nictocellulose membrane, and the membrane was incubated in 5% skim milk in PBS-T (PBS containing 0.05% Tween 20) for 1 hr at RT. Aß was detected using the anti-Aß anibody 6E10 (Covance, Princeton, NJ), an HRP-conjugated secondary antibody, and Amersham ECL^TM^ Western Blotting Detection reagents (GE Healthcare, UK).

### Electron microscopy

Transmission electron microscopy (TEM) was performed to observe Aß assembly following Aß incubation with liposomes. The samples negatively stained with 2% uranyl acetate on carbon-coated grids were examined under a JEM-1200EX transmission electron microscope (Tokyo, Japan) at an acceleration voltage of 80 kV.

### Statistical analysis

SAS (version 9.3) and GraphPad Prism 5 software were used. In LC-MS, statistical analysis by the Wilcoxon signed-rank test was performed for the comparison of three sets: sample *P1* (from the amyloid-free precuneus) and sample *P2* (from the amyloid-bearing precuneus), sample *C1* (from the calcarine cortex of the brains providing sample *P1*) and sample *P2*, sample *P2* and sample *C2* (from the calcarine cortex of the brains providing sample *P2*). The data are represented as mean ± SEM in lipid analysis.

## Results

### Aß assembly on SPM lipids prepared from the precuneus and the calcarine cortex

**Fig 2:**
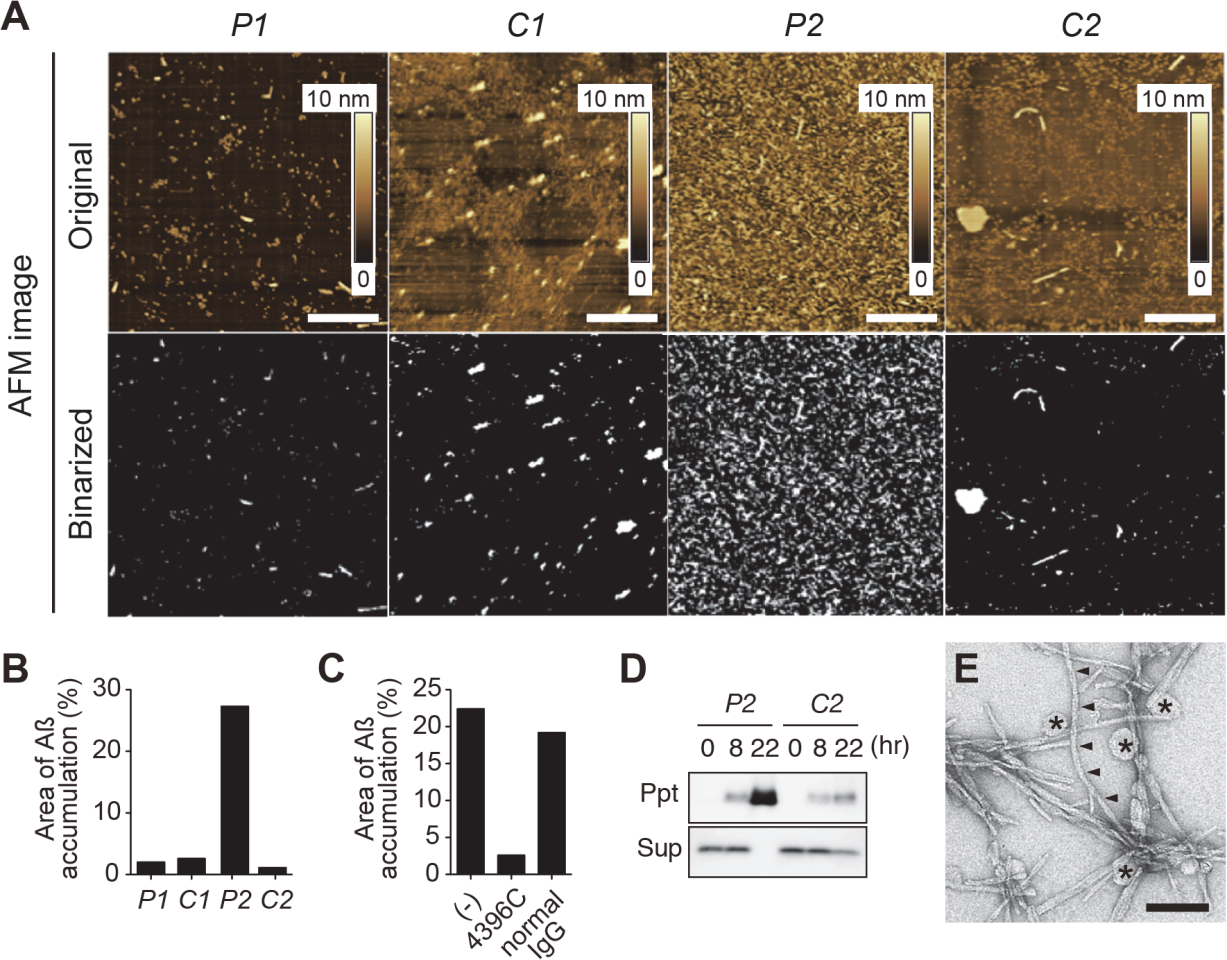
Aß assembly in the presence of the lipids extracted from SPMs isolated from autopsy brains. A, Representative original (*upper*) and binarized (*lower*) AFM images of Aß mixtures incubated on the reconstituted membranes prepared from extracted lipid samples; *P1* from the amyloid-free precuneus, *C1* from the calcarine cortex of the brain providing *P1*, *P2* from the amyloid-bearing precuneus, and *C2* from the calcarine cortex of the brain providing *P2*. The SPM sample containing same amount of proteins was used for each analysis. AFM images were taken after Aß incubation at 20 μM for 15 min. B, Area of Aß42 assembly on the membrane. C, Area of Aß42 assembly on the membrane of *P2* in the presence of 4396C or normal IgG. The mean of two independent experiments is shown in B and C. D, Western blots of insoluble (Ppt) and soluble (Sup) Aß42 following incubation with liposomes prepared from *P2* and *C2*. The insoluble Aß42 assemblies were solubilized in formic acid prior to Western blot analysis. The blot was immunostained with the anti-Aß antibody 6E10. E, TEM image of Aß42 mixtures incubated with liposomes prepared from *P2*. Typical amyloid fibrils with 6–10 nm diameter were observed (arrowheads). Asterisks indicate liposomes. Scale: 500 nm (A), 100 nm (E).

We prepared four lipid samples, including sample *P1*, from the amyloid-free precuneus (Fig. 1A); sample *C1*, from the calcarine cortex of the brains providing sample *P1* (Fig. 1B); sample *P2*, from the amyloid-bearing precuneus (Fig. 1C); and sample *C2*, from the calcarine cortex of the brains providing sample *P2* (Fig. 1D). We first tested whether sample *P2* were capable of accelerating Aß assembly by AFM (Fig. 1E), which enabled us to monitor the assembly of exogenously applied Aß42 on reconstituted bilayer membranes from a given lipid sample. Lipids were carefully extracted from the SPMs to thoroughly avoid contamination by pre-existing amyloid fibrils, which could facilitate Aß assembly. Remarkably, Aß assembly was accelerated on sample *P2* in an Aß concentration-dependent manner (Fig. 1F). We then compared the all four lipid samples; samples *P1*, *C1*, *P2* and *C2*. Notably, marked acceleration of Aß assembly was observed only on sample *P2* (Fig. 2A-B). Significantly, the Aß assembly was strongly inhibited by a monoclonal antibody against GAß (4396C) [9] (Fig. 2C), suggesting that the Aß assembly was through GAß generation on the membranes. To corroborate the Aß assembly in the presence of sample *P2*, we performed Western blot analysis of the incubated mixtures containing Aß42 and liposomes prepared from the extracted SPM lipids. The formation of insoluble Aß assemblies was markedly accelerated in the presence of sample *P2* (Fig. 2D). To morphologically characterize the Aß assemblies formed in the presence of sample *P2*, we performed transmission electron microscopy (TEM). We found typical amyloid fibrils with helical structure of 6–10 nm diameter [35] in the incubation mixture (Fig. 2E, arrowheads). Fibril-like thin structures were occasionally observed in the incubated mixture containing sample *C2*, but they were apparently different from those found in that containing sample *P2* (data not shown).

### Lipid composition of SPMs isolated from the precuneus and the calcarine cortex

**Fig 3:**
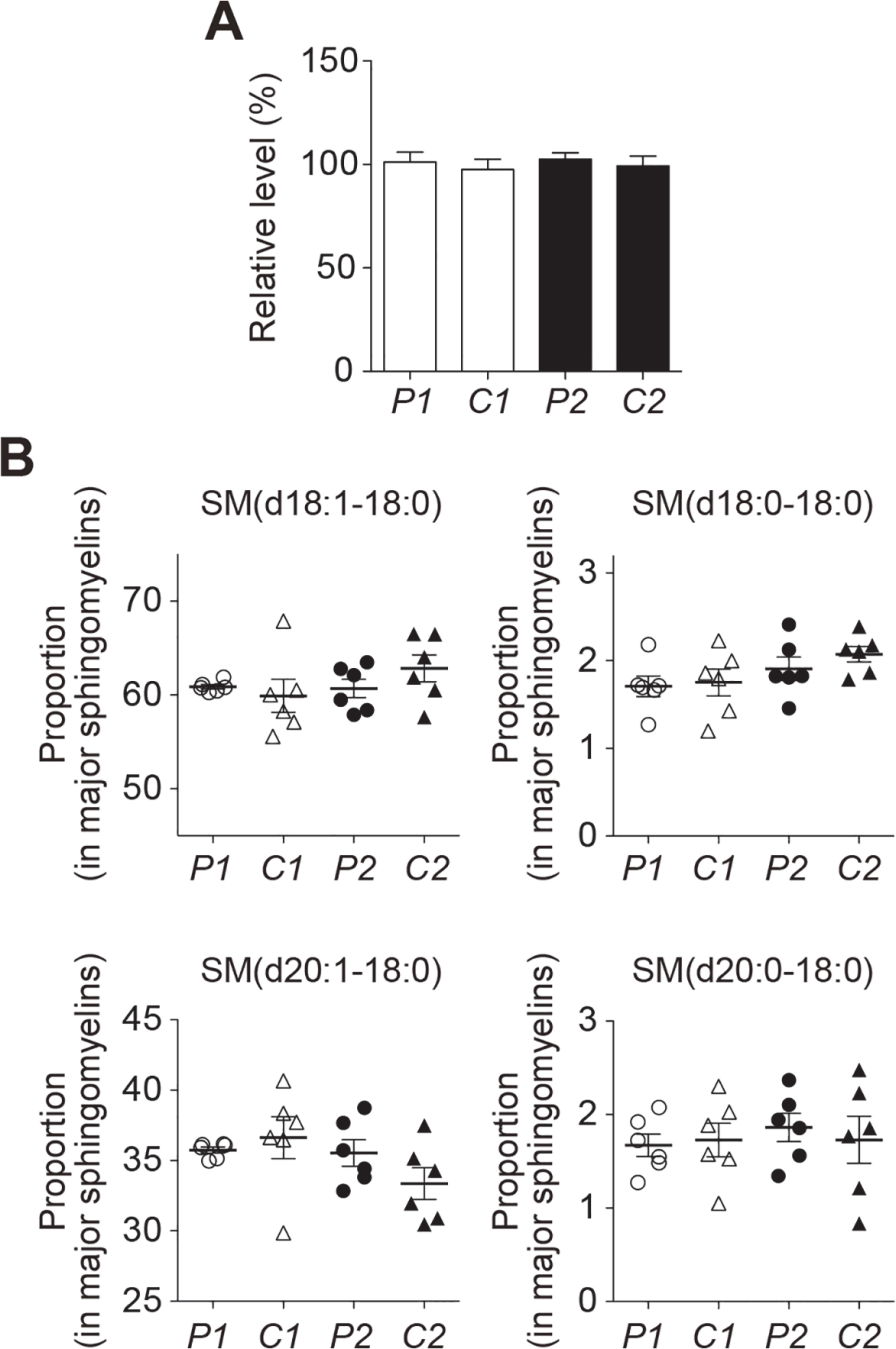
Lipid analyses of cholesterol and sphingomyelin of SPMs. A, Cholesterol level of SPMs. A, The cholesterol level of SPMs was determined by the cholesterol oxidase method using an Amplex-Red cholesterol assay kit. B, Composition of sphingomyelins of SPMs. Extracted sphingomyelins from SPMs were analyzed by reverse-phase LC-MS using a C30 column. The proportion of each sphingomyelin species containing diverse ceramide structures in four major sphingomyelins (containing d18 or d20 as the sphingoid base and C18:0 in the fatty acid chain) is expressed as mean ± SEM. *P1*, *C1*, *P2*, and *C2* indicate lipid samples extracted from SPMs of the amyloid-free precuneus, the calcarine cortex of the brain with the amyloid-free precuneus, the amyloid-bearing precuneus, and the calcarine cortex of the brain with the amyloid-bearing precuneus, respectively. The SPM sample containing same amount of proteins was used for each analysis.

**Fig 4:**
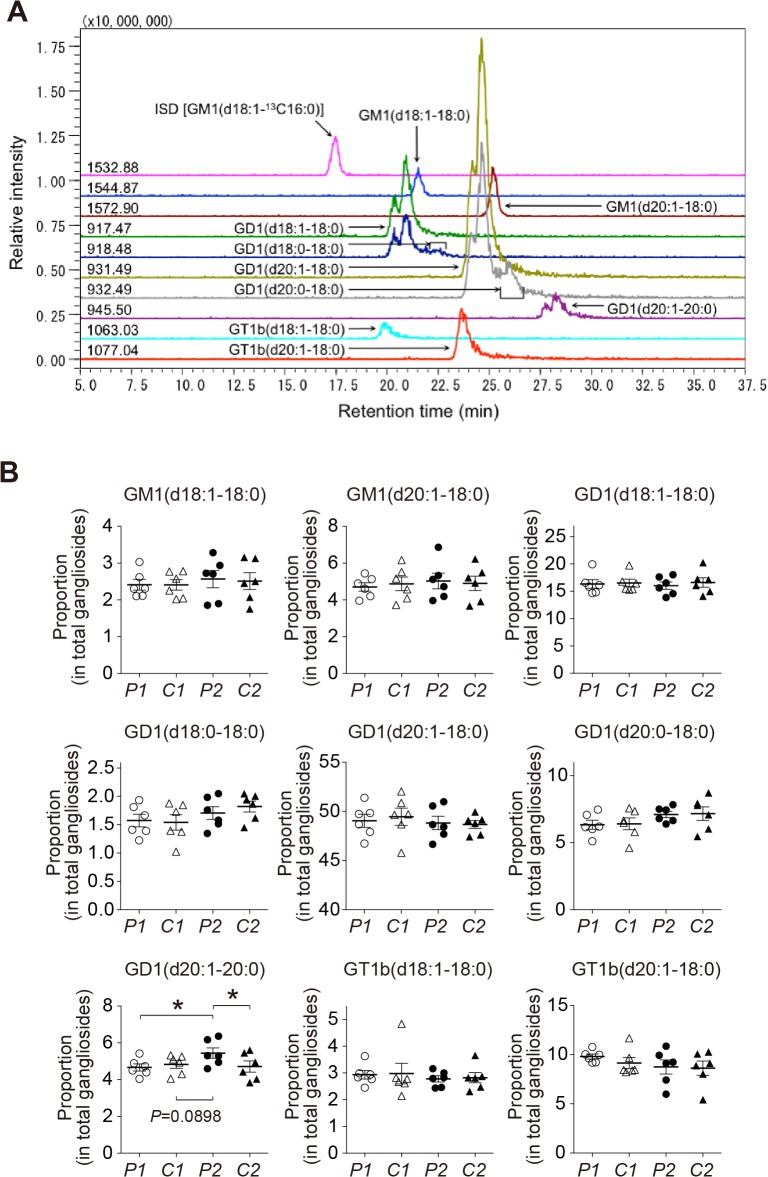
Increased proportion of GD1(d20:1–20:0) ganglioside species in the amyloid-bearing precuneus. A, Representative chromatograms of reverse-phase LC-MS of gangliosides using a C30 column. GM1(d18:1–^13^C16:0) [*m/z* 1532.88 (shown on the left side on the chromatogram)] was used as the internal standard (ISD). Ganglioside species containing diverse ceramide structures, such as d18:1 or d20:1 in the sphingoid base and 18:0 or 20:0 in the fatty acid chain, were separately detected in this system. The expression level of each ganglioside species was calculated by measuring each peak area above the baseline. B, Composition of gangliosides of SPMs. Gangliosides in the lipid samples extracted from SPMs were analyzed by reverse-phase LC-MS as indicated A. The proportion of each ganglioside species containing diverse ceramide structures in the detected gangliosides is expressed as mean ± SEM. *, *p*<0.05. *P1*, *C1*, *P2* and *C2* indicate lipid samples extracted from SPMs of the amyloid-free precuneus, the calcarine cortex of the brain with the amyloid-free precuneus, the amyloid-bearing precuneus, and the calcarine cortex of the brain with the amyloid-bearing precuneus, respectively.

**Fig 5:**
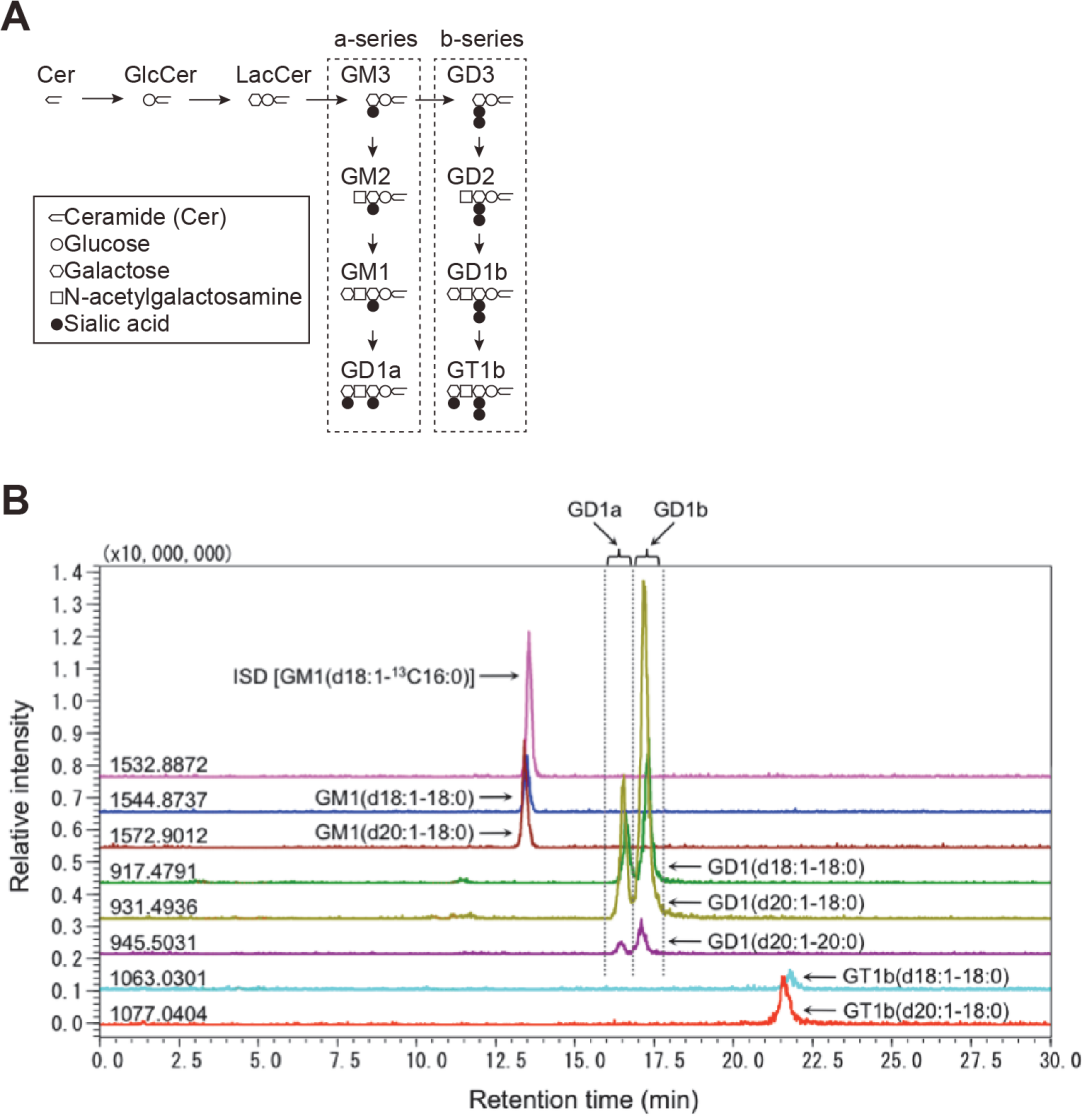
Analyses of gangliosides by normal-phase LC-MS with regard to the glycan structure of gangliosides. A, Scheme of synthesis pathway of *a*- and *b*-series of gangliosides. B, Representative chromatograms of normal-phase LC-MS of gangliosides using an NH_2_ column. GM1(d18:1–^13^C16:0) [*m/z* 1532.88 (shown on the left side on the chromatogram)] was used as ISD. In this system, *a*-series (GD1a) and *b*-series (GD1b) GD1-gangliosides were separately detected on the basis of the diversity of the glycan structure. The expression level of each ganglioside species was calculated by measuring each peak area above the baseline.

**Fig 6:**
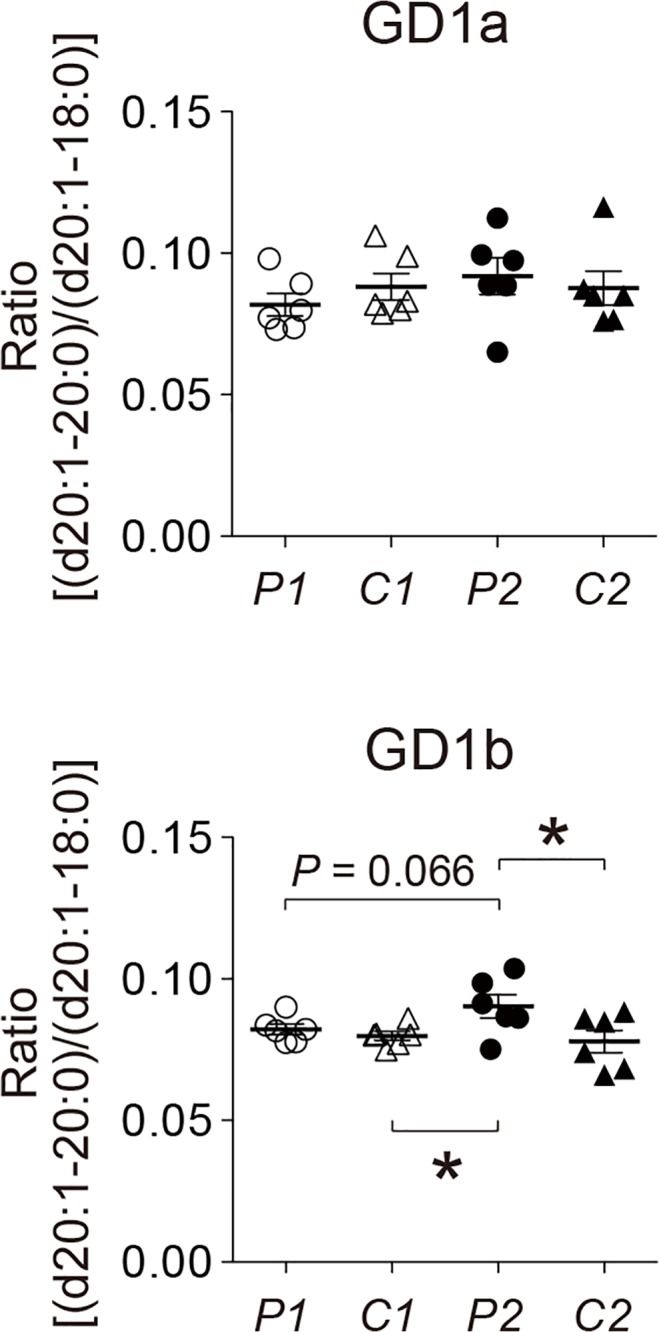
Increased ratio of level of (d20:1–20:0) to that of (d20:1–18:0) in GD1b-ganglioside in the amyloid-bearing precuneus. Ratios of the level of (d20:1–20:0) to that of (d20:1–18:0) in GD1a- and GD1b-gangliosides were calculated from the levels of *a*- and *b*-series of gangliosides obtained by normal-phase LC-MS using an NH_2_ column. The ratio is expressed as mean ± SEM. *, *p*<0.05. *P1*, *C1*, *P2* and *C2* indicate lipid samples extracted from SPMs of the amyloid-free precuneus, the calcarine cortex of the brain with the amyloid-free precuneus, the amyloid-bearing precuneus, and the calcarine cortex of the brain with the amyloid-bearing precuneus, respectively.

To gain insight into the mechanism underlying the acceleration of Aß assembly in the presence of sample *P2*, we searched for the difference in lipid composition among samples *P1*, *C1*, *P2*, and *C2*. We first measured the levels of cholesterol and sphingomyelin in these samples because both of these lipids potently facilitate GAß generation *in vitro* [20,21]. However, no significant difference was observed in these lipids among four samples (Fig. 3A-B). Then, we examined the levels of gangliosides using liquid chromatography-mass spectrometry (LC-MS). In the initial survey of the ganglioside profile by reverse-phase LC-MS using a C30 column in relation to the ceramide structure (Fig. 4A), the only remarkable finding was an increase in the proportion of GD1-ganglioside containing d20:1–20:0 as the ceramide structure [GD1(d20:1–20:0)] in sample *P2* compared with other samples (*p*<0.05 in comparison with sample *P1* and *C2*, and *p* = 0.0898 in comparison with sample *C1*) (Fig. 4B). We further characterized the increase in the GD1-ganglioside proportion in sample *P2*. We attempted to determine the levels of *a-* and *b*-series of GD1-ganglioside subspecies by LC-MS using an NH_2_ column with regard to the glycan structure of gangliosides (Fig. 5A-B). Consistent with an early study on the topographical predominance of *a-* and *b*-series of major ganglioside subspecies in the human brain [36], the mean proportions of GD1a-ganglioside subspecies were higher in the precuneus than in the calcarine cortex while those of GD1b-ganglioside subspecies were all lower in the precuneus than in the calcarine cortex (Table 2). However, the reversal of the predominance of the GD1b-ganglioside containing ceramide (d20:1–20:0) [GD1b(d20:1–20:0)] was exceptionally observed in sample *P2*, showing a mean proportion in the precuneus being higher than that in the calcarine cortex (Table 2). To validate the uniqueness of the increased proportion of GD1b(d20:1–20:0) in sample *P2*, we calculated the ratio of the level of GD1b(d20:1–20:0) to that of GD1b-ganglioside comprising the same sphingoid base but the more common fatty-acid chain GD1b(d20:1–18:0) [37]. Notably, the ratio in sample *P2* was significantly higher than those in other samples (*p*<0.05 in comparison with sample *C1* and *C2*, and *p* = 0.066 in comparison with sample *P1*), in contrast to that of GD1a-ganglioside (Fig. 6). The difference between sample *P2* and other samples was not conspicuous, as was observed by AFM. This was not surprising but rather anticipated, because the Aß assembly leading to amyloid deposition emerges at only restricted areas even for sample *P2* (Fig. 1C), thus likely affecting the whole-lipid composition of SPM to an only limited extent. Overall, we hypothesized that the alteration in the ratio of the level of GD1b(d20:1–20:0) to that of GD1b(d20:1–18:0) accounts for the accelerated Aß assembly in the precuneus.

**Table 2:** Imbalance of “*a*-series”-“*b*-series” composition in GD1(d20:1–20:0) in the brains harboring Aß deposition.

	*a*-series						*b*-series					
Ceramide	*P1*		*C1*	*P2*		*C2*	*P1*		*C1*	*P2*		*C2*
d18:1–18:0	6.58 (0.61)	>	5.69 (0.57)	6.91 (0.74)	>	5.62 (0.53)	14.64 (0.54)	<	15.92 (0.91)	14.45 (0.80)	<	15.92 (0.77)
d20:1–18:0	14.36 (0.97)	>	12.44 (1.38)	14.43 (1.31)	>	11.44 (1.03)	37.3 (1.76)	<	38.91 (1.68)	37.54 (1.78)	<	40.23 (0.96)
d20:1–20:0	1.18 (0.10)	>	1.10 (0.14)	1.34 (0.17)	>	0.98 (0.07)	3.07 (0.17)	<	3.1 (0.09)	3.39 (0.23)	**>**	3.14 (0.20)

Composition of GD1-gangliosides in relation to *a*- and *b*-series of gangliosides. The proportion of each ganglioside in detected gangliosides is expressed as mean with ± SEM in parentheses. *P1*, *C1*, *P2*, and *C2* indicate lipid samples extracted from SPMs of the amyloid-free precuneus, the calcarine cortex of the brain with the amyloid-free precuneus, the amyloid-bearing precuneus, and the calcarine cortex of the brain with the amyloid-bearing precuneus, respectively.

### Effect of the proportion of GD1b-ganglioside subspecies on the Aß assembly

**Fig 7:**
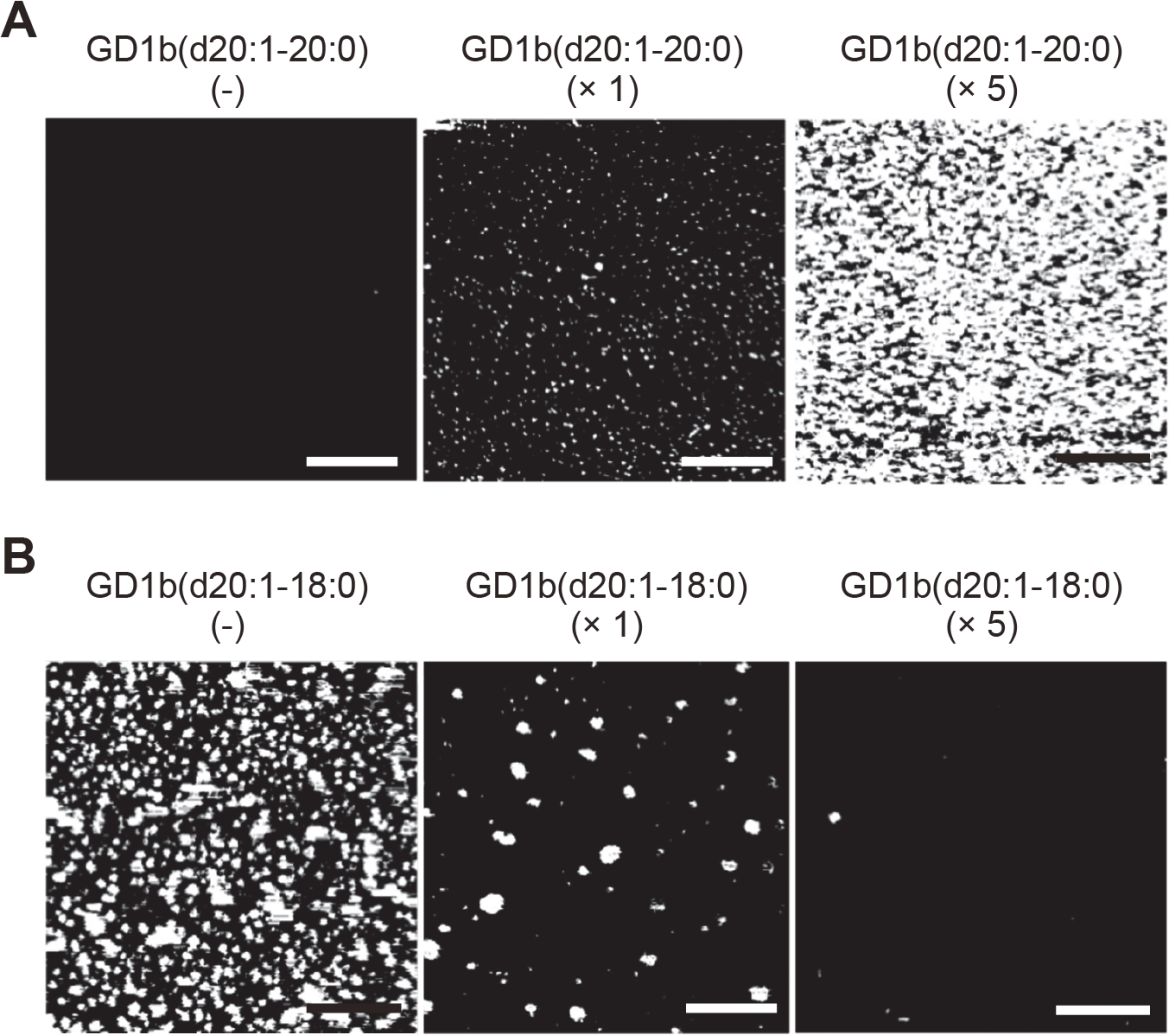
Effect of the alteration in the ratio of level of (d20:1–20:0) to that of (d20:1–18:0) in GD1b-ganglioside on Aß assembly. A, Addition of GD1b(d20:1–20:0) ganglioside to the lipids extracted from the amyloid-free precuneus significantly enhanced Aß assembly as determined by AFM. B, Addition of GD1b(d20:1–18:0) ganglioside to the lipids extracted from the amyloid-bearing precuneus significantly inhibited Aß assembly as determined by AFM. The subspecies of GD1b-ganglioside was purified from commercially available GD1b-gangliosides and added to the extent that its ratio equaled (*center*, × 1) or exceeded by four-fold (*right*, × 5) the original ratio in the lipids extracted from the amyloid-bearing precuneus. Scale bar, 500 nm.

To test the hypothesis mentioned above, we added GD1b(d20:1–20:0) or GD1b(d20:1–18:0), purified from commercially available porcine brain GD1b-gangliosides, to the lipids extracted from different sample sets. We then investigated the effect of the change in the ratio of the GD1b-ganglioside subspecies on Aß assembly. First, addition of GD1b(d20:1–20:0) to the lipids of sample *P1*, which increased the ratio of the level of GD1b(d20:1–20:0) to that of GD1b(d20:1–18:0) to mimic the condition of sample *P2*, dramatically accelerated Aß assembly on the reconstituted membranes (Fig. 7A). In contrast, addition of GD1b(d20:1–18:0) to the lipids of sample *P2*, which decreased the ratio of the level of GD1b(d20:1–20:0) to that of GD1b(d20:1–18:0) to mimic the condition of sample *P1*, significantly quenched the enhanced Aß assembly (Fig. 7B). These results indicate that an increase in the ratio of the level of GD1b(d20:1–20:0) to that of GD1b(d20:1–18:0) is a determinant for the enhancement of Aß assembly in the precuneus. Alternatively, addition of GD1b(d20:1–20:0) to the lipids of sample *C2*, which increased the ratio of the level of GD1b(d20:1–20:0) to that of GD1b(d20:1–18:0) to mimic the condition of sample *P2*, failed to enhance Aß assembly (data not shown).

## Discussion

Through liquid chromatography-mass spectrometry of the lipids extracted from SPMs isolated from the precuneus and the calcarine cortex of autopsy brains, one of the most vulnerable and the most resistant regions to amyloid depoistion, respectively, we identified an increase in the ratio of the level of GD1b-ganglioside containing C20:0 fatty acid to that containing C18:0 as a cause for the enhanced Aß assembly in the precuneus. Taken together with our previous finding that GAß facilitates Aß assembly into fibrils in the brain by acting as a seed [7,9], this study provides further evidence to support a hypothesis that sphingolipids play a critical role in AD development [38]. Also, to the best of our knowledge, this is the first study to show that an imbalance in fatty-acid-chain length of gangliosides in cellular membranes triggers a crucial pathogenic step in human diseases.

There may be a question of whether results obtained from autopsy brains represent secondary alterations but not primary events in the pathological process of the diseases. In this study, to detect early changes in membrane lipids that trigger but not are caused by amyloid deposition, we carefully collected brain specimens at very early stage of amyloid deposition. In the initial profiling of gangliosides of this study, the levels of major gangliosides, including GD1b-ganglioside, did not decrease in sample *P2* as was previously observed in AD brains [39–41] (data not shown). Thus, the alteration in the proportion of GD1b-ganglioside subspecies observed in this study was not likely attributable to amyloid deposition. Rather, the results of the addition of exogenous GD1b-gangliosides with different ceramide structure into samples *P1* and *P2* (Fig. 7) indicate that the altered expression of GD1b-ganglioside subspecies is a cause of enhanced Aß assembly in the precuneus.

At this point, it remains to be elucidated how Aß assembly was enhanced on the reconstituted membrane of lipid sample *P2*. The significant inhibition of Aß assembly by 4396C, a monoclonal antibody raised against GAß (Fig. 2C), suggested that Aß assembly on the reconstituted membrane is likely through GAß generation. In terms of the mechanism underlying enhanced GAß generation on cellular membranes, it was previously suggested that local membrane lipids, including cholesterol and sphingomyelin [20,21], provide favorable milieu for GAß generation probably through facilitation of GM1-ganglioside clustering [20]. However, no significant difference was observed in these lipids in this study (Fig. 3). Thus, further driving force likely exists to facilitate GAß generation in the brain. Although further studies are required, it is intriguing to assume that GAß generation in the membranes is enhanced in a particular glycolipid condition, including the altered expression of GD1b-ganglioside subspecies as was observed in this study. In this regard, it is interesting to note that GM1-ganglioside appearance can be modulated by local glycolipid environment [42,43], especially by the neighboring GD1b-ganglioside [44]. In this context, it is noteworthy that elimination of GD3-ganglioside synthase, causing deficiency of *b*-series of gangliosides, including GD1b-ganglioside, completely suppressed amyloid deposition in Alzheimer mouse model [45]. At this stage, it remains to be clarified how a longer chain of fatty acid of GD1b-ganglioside can be involved in promoting the segregation of GM1-ganglioside. Besides a possibility of direct association with GM1-ganglioside, GD1b-ganglioside with a longer hydrophobic chain may have a unique effect on lateral phase separation of GM1-ganglioside through interaction with coexisting lipids such as phosphatidylcholine harboring variable length of acyl chains [46,47].

In this study, we intend to search for the difference of lipid composition between precuneus and calcarine cortex, that is responsible for regional vulnerability/resistance to amyloid deposition. We have successfully identified imbalance of GD1b-ganglioside subspecies as a causative factor for the initiation of Aß assembly in the precuneus. However, addition of GD1b(d20:1–20:0) to the lipids of sample *C2*, which increased the ratio of the level of GD1b(d20:1–20:0) to that of GD1b(d20:1–18:0) to mimic the condition of sample *P2*, failed to enhance Aß assembly (data not shown). This result implys that the alteration in the ratio of the level of GD1b(d20:1–20:0) to that of GD1b(d20:1–18:0) alone is not sufficient for overcoming the regional barrier to amyloid deposition between the precuneus and the calcarine cortex. An intriguing and challenging question of why calcarine cortex is resistant to amyloid deposition still remains.

How do we rationalize the unique alterations in the ganglioside expression pattern in the SPMs isolated from the precuneus? The precuneus is a key region of the default mode network (DMN) [48], which is selectively and early impaired in AD [49,50]. Although the biological basis for the DMN at cellular level remains unknown, the regulatory mechanism of synaptic construction, including lipid composition, may be different from that in the DMN in other regions. Indeed, the characteristic features of cytoarchitectonic structures and synaptic connectivity were found in the precuneus [48]. Notably, the alteration in the GD1b-ganglioside expression pattern observed in this study showed concordance with the change in the expression patterns of major gangliosides, including GD1b-ganglioside, with age [51–53]. Thus, ageing-associated changes in ganglioside expression pattern may be accelerated somehow in the precuneus of individuals at risk of developing AD. It is also noteworthy that synthase for ceramide containing long chain fatty acid is selectively upregulated in the early stage of AD [54]. Thus, it may be challenging and intriguing to explore the specificity of the precuneus from a viewpoint of the expression of different ceramide synthases in future studies.

It remains unknown whether the imbalance in the fatty-acid-chain length in gangliosides can be generally causative for amyloid deposition beyond the precuneus. Nevertheless, our results indicate that this imbalance is a strong *bona fide* driving force for initiating Aß assembly in the precuneus. As suggested by accumulating evidence, sphingolipids, including complex gangliosides, may be critical players in AD [38]. In addition, it should be elucidated in the future studies whether the change in the ganglioside compostion in the precuneus induce various neurobiological effects beyond initiation of Aß assembly.
